# Enhanced expression of a 35 kDa fragment of inter-alpha-trypsin inhibitor H4 in sera of healthy pregnant women and patients with hydatidiform mole

**DOI:** 10.1186/2050-7771-1-19

**Published:** 2013-05-15

**Authors:** Emida Mohamed, Jaime Jacqueline Jayapalan, Puteri Shafinaz Abdul-Rahman, Siti Zawiah Omar, Onn Haji Hashim

**Affiliations:** 1 Department of Molecular Medicine, Faculty of Medicine, University of Malaya, Kuala Lumpur, Malaysia; 2 University of Malaya Centre for Proteomics Research, Faculty of Medicine, University of Malaya, Kuala Lumpur, Malaysia; 3 Department of Obstetrics and Gynaecology, Faculty of Medicine, University of Malaya, Kuala Lumpur, Malaysia

## Abstract

**Background:**

Accumulated data from previous studies appear to suggest a link between the overexpression of a 35 kDa fragment of serum inter-alpha-trypsin inhibitor H4 (ITIH4) with cancers that are associated with up-regulated levels of oestrogens. The truncated fragment was postulated to be a product of oestrogen-induced action of kallikrein on native ITIH4. The present lectin-based proteomic analyses were performed to assess the specificity of the 35 kDa fragment of ITIH4 as a potential cancer biomarker and determine whether it was also overexpressed in the sera of cancer-negative pregnant women who are known to have high levels of plasma oestrogens.

**Results:**

Our results demonstrated that the 35 kDa fragment of ITIH4 was overexpressed in healthy pregnant women and patients with hydatidiform mole, relative to the controls. The serum oestradiol levels of both groups of pregnant subjects were also confirmed to be higher than those of the control women who were not pregnant.

**Conclusions:**

Overexpression of the 35 kDa fragment of ITIH4 was not restrictive to patients with cancers but also occurred in women who were pregnant and those diagnosed with hydatidiform mole. Our data implicate the limitation of the 35 kDa ITIH4 fragment as a cancer biomarker and its correlation with serum oestrogen levels.

## Background

Inter-alpha-trypsin inhibitor heavy chain 4 (ITIH4) is a member of the inter-alpha-trypsin inhibitor (ITI) family of hepatic origin [1,2]. It is the only member of the ITI family which harbours a kallikrein-released bradykinin-like domain in its C-terminal sequence [3], making it plasma kallikrein sensitive [4-6]. Trace amounts of plasma kallikrein have been shown to cleave ITIH4 to yield two fragments, i.e., a 35 kDa C-terminal polypeptide and an 85 kDa N-terminal fragment [7]. The 35 kDa ITIH4 fragment, which is *O*-glycosylated [3,8], is assumed to remain intact. However, the 85 kDa ITIH4 fragment is further cleaved to produce an N-terminal 57 kDa fragment and a putative 28 kDa fragment. The latter is believed to be further processed by protease(s) to generate smaller fragments [8].

We have previously analyzed the expression of the 35 kDa ITIH4 fragment in groups of patients with nine different types/subtypes of cancers using the gel-based proteomics approach and a lectin that binds to *O*-glycosylated proteins [9-12]. Whilst the serum ITIH4 fragment was demonstrated to be overexpressed in patients with endometrial cancer, ovarian cancer (germ-line and epithelial ovarian carcinoma) and breast cancer compared to the control subjects, its levels were not significantly different in sera of patients with nasopharyngeal carcinoma, osteosarcoma (localized disease), cervical cancer (squamous cell cervical carcinoma and cervical adenocarcinoma) and prostate cancer [9-11]. In the latter cancer, however, significantly enhanced levels of a similar ITIH4 fragment was later detected in the urine of the patients [12].

One of the obvious differences between the types of cancers that are associated with overexpression of the 35 kDa ITIH4 fragment with those that did not is that the former also appears to be associated with the up-regulated levels of serum oestrogens [13,14]. This is suggestive of the potential use of the ITIH4 fragment as a complementary biomarker for oestrogen-related cancers. However, enhanced blood oestrogen levels are not restrictive to patients with cancer but also occur in healthy women who are pregnant as well as those detected with benign tumours. Hence, the present study was carried out to assess the specificity of the 35 kDa ITIH4 fragment as a potential biomarker for cancer and whether it was also overexpressed in two groups of cancer-negative women who were pregnant and are known to have high levels of plasma oestrogens. The first group of subjects comprised healthy pregnant women, whilst the second were those who were diagnosed with hydatidiform mole, a type of gestational trophoblastic disease that is associated with a rare mass or growth that forms inside the uterus at the beginning of a pregnancy.

## Results

### Determination of serum oestradiol levels

**Figure 1:**
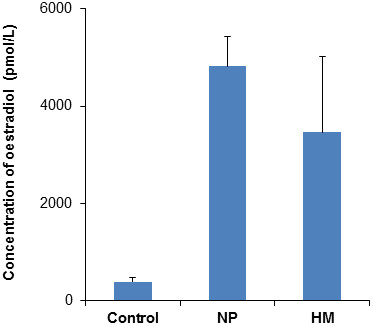
**Serum oestradiol levels.** Serum samples were subjected to quantitative measurements of oestradiol levels as described in the Methods section. All values are presented as mean ± SEM.

The present study was performed to assess the specificity of the 35 kDa fragment of ITIH4 as a potential cancer biomarker and determine whether it was overexpressed in the sera of pregnant women (n = 20) and patients with hydatidiform mole (n = 20), who are known to have higher levels of serum oestrogens, relative to normal healthy subjects (n = 20). To confirm for the altered levels of serum oestrogens in the women who were pregnant and those with hydatidiform mole compared to the controls, their serum samples were analysed for their oestradiol levels as described in the Methods section. Figure 1 demonstrates that the mean levels of serum oestradiol were significantly higher in the women who were pregnant (4816 ± 610 pmol/L) and those with hydatidiform mole (3468 ± 1955 pmol/L), compared to the controls (393 ± 91 pmol/L).

### Two-dimensional electrophoretic profiles of serum *O*-glycosylated proteins

In this study, the expression of a 35 kDa ITIH4 fragment, which is *O*-glycosylated, was analysed using an earlier established method involving two-dimensional gel electrophoresis (2-DE), western blotting and the use of champedak galactose binding (CGB) lectin to detect *O*-glycosylated proteins [9-12]. The CGB lectin was chosen on the basis of its specific interaction with *O*-glycans [15,16].

**Figure 2:**
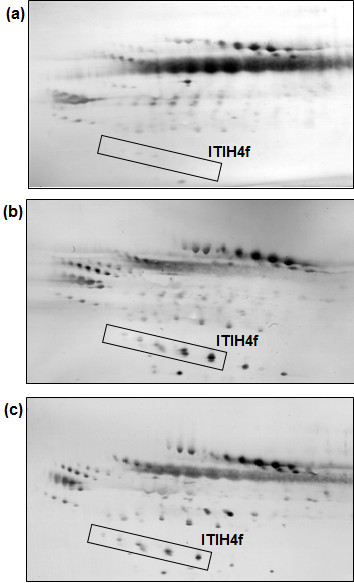
**Detection of 2-DE separated ITIH4 fragment on nitrocellulose membrane.** Neat serum samples were subjected to 2-DE, western blotting and detection with enzyme-conjugated CGB lectin. Panel (**a**) demonstrates a typical profile of healthy non-pregnant women controls, while panels (**b**) and (**c**) are representative profiles of healthy pregnant women and patients with hydatidiform mole, respectively. The cleavage fragment of ITIH4 (ITIH4f) appears to be overexpressed in all subjects’ profiles relative to the controls. For all panels, the acidic side of the blot is to the left and relative molecular mass declines from the top.

Figure 2 demonstrates representative 2-DE serum *O*-glycosylated protein profiles of (a) control subjects, (b) healthy pregnant women and (c) patients with hydatidiform mole that were resolved using enzyme-conjugated CGB lectin. A total of six clusters of *O*-glycosylated serum proteins were consistently detected in all the lectin-developed profiles, whilst several others had low rates of presence in the 2-DE gels. Like most other serum glycopeptides, the 35 kDa cleavage fragment of ITIH4 were separated into a cluster of five isoform spots because of its heterogeneous glycan structures. Both groups of women sera that were studied demonstrated enhanced expression of the 35 kDa ITIH4 fragment cluster relative to that of the non-pregnant healthy women controls.

### Identification of ITIH4 fragment spot cluster

The identity of ITIH4 fragment spot cluster was confirmed by subjecting the serum protein to MALDI ToF/ToF analysis and search of the Swiss-Prot database (Table 1). Whilst the database search indicates that the spot cluster of interest was that of ITIH4, the approximate experimental molecular weight that was derived from migration of the protein in the 2-DE protein profiles suggests that it was a truncated 35 kDa fragment of the polypeptide.

**Table 1:** Confirmation of the identity of serum ITIH4 by MS/MS

**Spot/ Cluster ID**	**Matched protein identity**	**Swiss-prot accession number**	**Theore-tical Mass (Da)**	**Theore-tical pI**	**Mascot score**	**No. of peptides matched**	**Sequence coverage (%)**
ITIH4	Inter-alpha-trypsin inhibitor heavy chain H4^**(a)**^	Q14624	103293	6.51	169	2	3

^(a)^ITIH4 spot cluster was a fragment based on its estimated experimental molecular weight.

### Image analysis of *O*-glycosylated ITIH4 fragment

**Figure 3:**
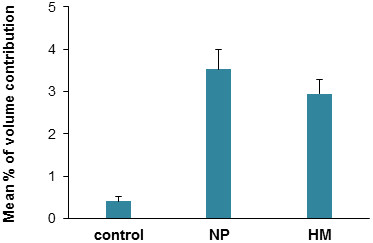
**Expression of 35 kDa ITIH4 fragment in sera of women with normal pregnancy and hydatidiform mole.** Analysis was performed on sera of healthy non-pregnant women controls (n = 20) and healthy pregnant women (NP; n = 20) and those with hydatidiform mole (HM; n = 20). Expression of ITIH4 fragment was analysed in terms of the percentage of volume contribution, which refers to the spot volume of the glycoprotein expressed as a percentage of the total spot volume of all detected serum glycoproteins. All values are presented as mean ± SEM. Data were analyzed by Student’s t-test and a p value of less than 0.01 was considered significant. Asterisks denote significantly different values as compared to controls.

When the 35 kDa serum ITIH4 fragment cluster was analysed by densitometry, significant up-regulated expression was detected in the women who were pregnant (+8.7-fold; p = 0.0002) as well as in the patients with hydatidiform mole (+5.6-fold; p < 0.0001) relative to the controls (Figure 3). This is comparable with values that were obtained for patients with endometrial cancer, germ-line and epithelial ovarian cancers, and breast cancer [9,10].

## Discussion

Our previous accumulated studies have demonstrated the overexpression of a 35 kDa ITIH4 fragment selectively in cancers associated with elevated oestrogen levels, including cancers of the breast, endometrium, ovary and prostate but not in nasopharyngeal carcinoma, osteosarcoma and cervical cancer. To investigate the possibility that the 35 kDa ITIH4 fragment may also be enhanced in non-cancer conditions that are associated with up-regulated levels of oestrogens, analysis was extended to include two groups of non-cancer patients with similar hormonal dysregulation. In the present study, two groups of women with different pregnancies and with increased levels of oestrogens were chosen. The first group comprised healthy women who were pregnant, which represents a normal condition, while the second involved patients with hydatidiform mole and represents a benign condition.

The maternal levels of circulating oestrogens increase continuously throughout a normal pregnancy, as it is required to support foetal development [17-19]. This is also seen when the healthy women subjects who were pregnant were analysed for their serum oestrogens in the present study. Similarly, women with hydatidiform mole have also been reported to have plasma oestrogen levels as high as those with normal pregnancy [20] and this is also reflected from their serum oestradiol values that were determined in this study.

**Figure 4:**
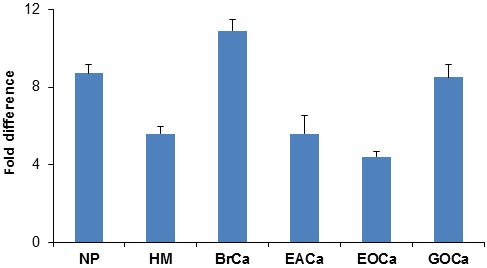
**Relative expression of ITIH4 fragment in serum samples.** Fold-differences calculated for healthy pregnant women (NP) and those with hydatidiform mole (HM) are values relative to the expression of ITIH4 fragment in the control subjects. Values for patients with breast cancer (BrCa), endometrial cancer (EACa), epithelial ovarian cancer (EOCa) and germ-line ovarian cancer (GOCa) were those that had been previously reported [9,10].

When sera of both groups of pregnant subjects were analysed using an earlier established CGB lectin-based electrophoretic approach in the present study, the expression of the 35 kDa ITIH4 fragment was prominently up-regulated compared to the controls. The fold differences obtained for the healthy pregnant women and patients with hydatidiform mole were comparable with those that were earlier established for patients with endometrial cancer, germ-line and epithelial ovarian cancers, and breast cancer (Figure 4). Hence, these results, when taken together with the data of the previous studies, generally indicate that the overexpression of the 35 kDa ITIH4 fragment was not exclusive to oestrogen-related cancers but also occurs in normal pregnancy and benign conditions.

The marked difference in the expression of the 35 kDa ITIH4 cleavage fragment detected in the various subjects with enhanced oestrogen levels may be attributed to the increased cleavage of ITIH4 by elevated levels of circulating kallikreins in the serum. The idea that the abundance of the ITIH4 fragment is linked to high amounts of serum kallikreins is derived from previous reports demonstrating overexpression of members of the kallikrein family in cancers of the breast, ovary and endometrium [21-23]. This is not surprising as kallikreins are known to be expressed in hormone-dependant tissues such as the breast and ovary [24]. In addition, the expression of the kallikrein genes have been shown to be regulated by steroid hormones (including oestrogens) in cancer cell lines [22,25]. As the ITIH4 protein is kallikrein-sensitive, there is likelihood that the overexpression of kallikrein may lead to increased cleavage of serum ITIH4 which in turn led to the enhanced liberation of its 35 kDa C-terminal fragment. In support of this correlation is a study conducted by Gangadharan *et al.*[26], which showed that the down-regulation of kallikrein in patients with hepatic cirrhosis resulted in low abundance of the ITIH4 fragments including the 35 kDa fragment.

## Conclusions

The data of the present study, when taken together with those of our previous reports, suggest that overexpression of the 35 kDa fragment of ITIH4 is oestrogen-related and occurs in patients with selective cancers, hydatidiform mole as well as healthy women who are pregnant. This implicates the limitation of the ITIH4 fragment as a biomarker for cancer.

## Methods

### Collection of serum samples

Serum samples were collected with patients’ consent at the Obstetrics and Gynaecology ward, University of Malaya Medical Centre (UMMC), Kuala Lumpur in accordance to a protocol that was approved by the Medical Ethics Committee of the centre. Samples from groups of women who were pregnant (n = 20) and patients with hydatidiform mole (n = 20) were collected in their first trimester of pregnancy. For comparison, sera from normal healthy non-pregnant women (n = 20) were obtained from age-matched volunteers (range of 21–45 years). Blood samples were collected in fresh 1.5 ml BD vacutainers (Becton, Dickinson & Co, Franklin Lakes, New Jersey, USA) and were centrifuged at 3000 g for 10 min (Centrifuge 5403, Eppendorf, Hamburg, Germany). Serum was collected and stored in aliquots of 100 μl at −80° until used.

### Biochemical tests

Quantitative measurements of oestradiol in the subjects’ serum samples were performed according to the manufacturer’s instructions using the ADVIA Centaur and ADVIA Centaur XP Systems (Siemens Medical Solutions Diagnostics, Tarrytown, USA). Values of serum oestradiol are expressed in mean ± SEM.

### 2-DE

2-DE was performed as previously described [27] using approximately 800 μg protein. Neat serum samples were initially incubated in 2% v/v IPG sample buffer pH 4–7, containing 9 M urea, 60 mM DTT, and 0.5% v/v Triton X-100 at room temperature for 30 min. They were then incubated in a rehydration solution containing 8 M urea, 0.5% v/v IPG buffer, 0.5% v/v Triton X-100 for another 30 min before incubating with rehydrated IPG Immobiline Drystrips pH 4–7, 11 cm (GE Healthcare, Uppsala, Sweden) overnight. The strips were subjected to isoelectric focusing using the Multiphor Flatbed electrophoresis system (GE Healthcare, Uppsala, Sweden) for a total duration of 15 kV/h (Phase 1: 300 V, 2 mA, 5 W, 30 min; Phase 2: 3500 V, 2 mA, 5 W, ∼4–4.5 h). Focused strips were equilibrated in 1.5 M Tris–HCl (pH 8.8) solution containing 6 M urea, 2% w/v SDS, 30% v/v glycerol, and 0.06 M DTT for 15 min on a UNIMAX 2010 platform shaker and further incubated in a similar equilibration solution but containing 4.5% v/v iodoacetamide instead of DTT for another 15 min. The equilibrated strips were overlaid onto 8-18% gradient polyacrylamide gels and electrophoresis was performed following an optimized protocol (Phase 1: 50 V, 40 mA, 25 W for 30 min; Phase 2: 600 V, 40 mA, 25 W for 1–2 h) using the SE 600 Ruby Electrophoresis System and Power Supply-EPS601 (GE Healthcare, Uppsala, Sweden).

### Western blotting and detection of *O*-glycosylated proteins

The 2-DE-separated proteins were transferred electrophoretically onto nitrocellulose (NC) membranes (0.45 mM; Whatman, Dassel, Germany) using the NovaBlot Kit of the Multiphor™ II Flatbed System (GE Healthcare, Uppsala, Sweden) for 2 h at a constant current of 0.8 mA/cm^2^ gel. Detection of transferred *O*-glycosylated serum proteins was performed using the CGB lectin that was affinity purified and characterized for its specificity to *O*-glycans using methods that were previously reported [28]. The lectin was then conjugated to horseradish peroxidase before being used to probe for *O*-glycopeptides on the NC membranes. The membranes were finally developed by means of a colorimetric reaction using diamino-benzoic acid as substrates.

### Mass spectrometry

Protein spots of interest were carefully excised from the blot for the subsequent on-membrane trypsin digestion according to method that was previously described [29]. The MS/MS analysis was performed using the 4800 Plus MALDI ToF/ToF analyzer (Applied Biosystems, Foster City, CA, USA).

### Database search

Identification of proteins was performed using the MASCOT search engine [30]. The MS data obtained was searched against *Homo sapien* entries in the Swiss-Prot database (Last update: February 15, 2012, containing 535248 sequences) according to the following selection parameters: enzyme - trypsin, missed cleavage - 1, variable modification - 2; i) carbamidomethylation of cysteine and ii) oxidation of methionine, MS precursor ion mass tolerance - 100 ppm, MS/MS fragment ion mass tolerance - 0.2 Da, and inclusion of monoisotopic masses only.

### Image analysis

CGB lectin-probed NC blots were scanned using Imaging Densitometer GS690 (Bio-Rad Laboratories, Hercules, California, USA). Expression of ITIH4 fragment was analysed in terms of the percentage of volume contribution, which refers to the spot volume of the glycoprotein expressed as a percentage of the total spot volume of all detected serum glycoproteins, using the Image Master 2D Platinum software, version 7.0 (GE Healthcare Biosciences, Uppsala, Sweden). Cut-off parameters were: Smooth – 2; Saliency – 1; Min area – 5. Data expressed in this manner are independent of variations attributed to protein loading and staining.

## Competing interests

The authors declare no conflict of interest.

## Authors’ contributions

EM carried out the studies, analyzed the data and drafted the manuscript. JJJ performed the mass spectrometry analysis and database search. PSAR helped in data analysis and interpretation. SZO provided serum samples and clinical input. OHH conceived the experiments, interpret the data and critically revised the manuscript. All authors have read and approved the final manuscript.
